# Transcriptome analysis of aphid-resistant and susceptible near isogenic lines reveals candidate resistance genes in cowpea (*Vigna unguiculata*)

**DOI:** 10.1186/s12870-022-04021-w

**Published:** 2023-01-11

**Authors:** Jacob R. MacWilliams, Paul D. Nabity, Kerry E. Mauck, Isgouhi Kaloshian

**Affiliations:** 1 Graduate Program in Biochemistry and Molecular Biology, University of California Riverside, Riverside, CA 92521 USA; 2 Department of Botany and Plant Sciences, University of California Riverside, Riverside, CA 92521 USA; 3 Institute for Integrative Genome Biology, University of California Riverside, Riverside, CA 92521 USA; 4 Department of Entomology, University of California Riverside, Riverside, CA 92521 USA; 5 Department of Nematology, University of California Riverside, Riverside, CA 92521 USA

**Keywords:** Herbivory, QTL, Antibiosis, Antixenosis, Legume

## Abstract

**Background:**

Cowpea (*Vigna unguiculata*) is a crucial crop for regions of the world that are prone to both heat and drought; however, the phytotoxic cowpea aphid (*Aphis craccivora)* impairs plant physiology at low population levels. Both antibiotic and antixenotic forms of resistance to the aphid have been mapped to two quantitative trait loci (QTLs) and near isogenic lines (NILs). The molecular mechanism for this resistance response remains unknown.

**Results:**

To understand the genes underlying susceptibility and resistance, two cowpea lines with shared heritage were infested along a time course and characterized for transcriptome variation. Aphids remodeled cowpea development and signaling relative to host plant resistance and the duration of feeding, with resource acquisition and mobilization determining, in part, susceptibility to aphid attack. Major differences between the susceptible and resistant cowpea were identified including two regions of interest housing the most genetic differences between the lines. Candidate genes enabling aphid resistance include both conventional resistance genes (e.g., leucine rich repeat protein kinases) as well as multiple novel genes with no known orthologues.

**Conclusions:**

Our results demonstrate that feeding by the cowpea aphid globally remodels the transcriptome of cowpea, but how this occurs depends on both the duration of feeding and host-plant resistance. Constitutive expression profiles of the resistant genotype link aphid resistance to a finely-tuned resource management strategy that ultimately reduces damage (e.g., chlorosis) and delays cell turnover, while impeding aphid performance. Thus, aphid resistance in cowpea is a complex, multigene response that involves crosstalk between primary and secondary metabolism.

**Supplementary Information:**

The online version contains supplementary material available at 10.1186/s12870-022-04021-w.

## Introduction

Cowpea (*Vigna unguiculata*) is a leguminous grain crop grown widely in semi-arid and tropical regions, with nearly half of all production taking place in sub-Saharan Africa. In 2018, over 6 million tons were produced in this region of the world alone [1]. Cowpea is important as a grain crop because of its high nutritional value and ability to withstand drought and heat stress [2]. Cowpea also has a high protein content, around 30%, and is an important source of vitamins and minerals [3].

Despite its ability to tolerate abiotic stress, cowpea is vulnerable to attack by yield-reducing biotic agents, including insects. One insect, the cowpea aphid (*Aphis craccivora*), is especially devastating because its subtle feeding behavior minimizes detection by the host plant yet results in yield loss [3, 4]. Cowpea aphids ingest phloem sap through piercing-sucking mouthparts, called stylets, which function like flexible needles to navigate between cells with minimal mechanical damage [5, 6]. Cowpea aphids also deposit saliva containing sheath proteins and effector molecules along the feeding path to ease stylet passage and suppress host-defense responses [7, 8]. Once the aphids reach the phloem they can engage in feeding (sap uptake) for prolonged durations.

Yield losses due to cowpea aphid can exceed over 50% of the grain yield [3]. Pesticides are effective in controlling the aphid and limiting spread. However, in sub-Saharan Africa, the economic and environmental costs of pesticides, including loss of beneficial insects, reduce the appeal and efficacy of chemical control methods. Because of these limiting factors, other strategies have been pursued to control cowpea aphid impacts, with breeding for host-plant resistance becomingthe predominant attractive, affordable, and sustainable option [9–11]. In response to this need, numerous cowpea genetic lines have been screened against aphids to identify germplasm that reduces aphid growth and/or displays tolerance (e.g., little to no chlorosis, necrosis, and leaf twisting, or pseudogalling in response to aphid feeding) [11, 12]. One of the most promising sources of cowpea aphid resistance was identified in an African breeding line (IT97K-556–6) [12]. The resistance source was crossed with the susceptible California Blackeye cultivar 27 (CB27) generating recombinant inbred lines (RILs). This RIL population underwent a field-based screen for aphid induced damage and the resistance was mapped to two quantitative trait loci (QTLs), a major QTL, *QAC-vu7.1,* and a minor QTL, *QAC-vu1.1* [10]. The IT97K-556–6 line was crossed to and repeatedly backcrossed with California Blackeye cultivar 46 (CB46) to generate resistant near-isogenic line (NIL) CB77 [10, 13].

The first exploration of the phenotype of the NIL resistance mechanism reported a lack of aphid damage symptoms after two weeks of infestation [14]. In contrast, susceptible plants (e.g. cowpea line CB46), show visible damage symptoms as early as a week after infestation with as few as 15 aphids (Fig. S1). Multiple no-choice assays showed that both aphid survival and fecundity are reduced on the resistant cowpea (Fig. S2), identifying antibiosis as the main mechanism of resistance. Antixenosis was identified as a secondary resistance mechanism through use of the electrical penetration graphing technique (EPG) and aphid choice assays. Cowpea aphids were deterred by the resistant NIL before phloem access and suffered reduced reproduction when forced to feed on the resistant NIL as the sole food source [14].

The cowpea resistance (*R*) loci, QTL, *QAC-vu7.1,* and QTL, *QAC-vu1.1,* encompass large genomic regions and the identity of the gene(s) contributing to this resistance remains unknown [10]. Although multiple genes conferring resistance to aphids have been identified in different plant species, to date only three of these *R* genes have been cloned [15, 16]. The first cloned aphid resistance gene was the tomato (*Solanum lycopersicum*) *Mi-1.2* gene, originating from the wild tomato species *Solanum peruvianum.* The *Mi-1.2* gene confers resistance against the potato aphid (*Macrosiphum euphorbiae*) [17, 18]. Another cloned *R* gene is *Vat* from melon (*Cucumis melo*) that confers resistance to certain genotypes of the cotton-melon aphid (*Aphis gossypii*) [19]. Both *R* genes encode classical R proteins with coiled-coil NB-LRR (CC-NB-LRR; CNL) domains, and both genes confer resistance to other pest species. Whereas *Mi-1.2* also confers resistance to three species of root-knot nematodes (*Meloidogyne arenaria, M. incognita and M. javanica*), whiteflies (*Bemisia tabaci*), and psyllids (*Bactericera cockerelli*) [20–23], the *Vat* gene alters feeding behaviors of the cotton-melon aphid thereby indirectly reducing virus transmission [24]. The most recently identified *R* gene for aphids is the Arabidopsis (*Arabidopsis thaliana*) *SIEVE ELEMENT-LINING CHAPERONE1 (SL1)* gene, which encodes a small heat shock like protein and confers resistance to the green peach aphid (*Myzus persicae*) by lining the sieve elements and obstructing the ability of the aphid to feed [15, 25].

To better understand the genetic factors underlying resistance present in cowpea CB77, we profiled gene expression over a time course of aphid damage using RNAseq. This time course included time points capturing early responses (1-day) and late responses following chronic damage (6-day). The early time point was chosen to elucidate responses underlying cowpea aphid non-preference for resistant cowpea, identified previously by EPG recordings and choice assays [14]. The later time point captures gene expression responses during prolonged aphid feeding, which may provide insight into the mechanism of antibiosis (reduced population growth) seen previously. For each time point, we (1) identified differentially expressed genes (DEGs) in resistant and susceptible cowpea through multiple contrasts; (2) identified patterns of gene expression through expression of similar type gene clusters; and (3) identified most likely candidates for the resistance conferred by matching gene expression patterns to regions of interest where the genotypes have the majority of their genomic differences. The outputs of this study will aid future endeavors to develop more endogenously resistant cowpea lines and contribute to our knowledge of resistance mechanisms against phloem-feeding insects.

## Results

### Aphid feeding remodels the cowpea transcriptome

**Fig. 1:**
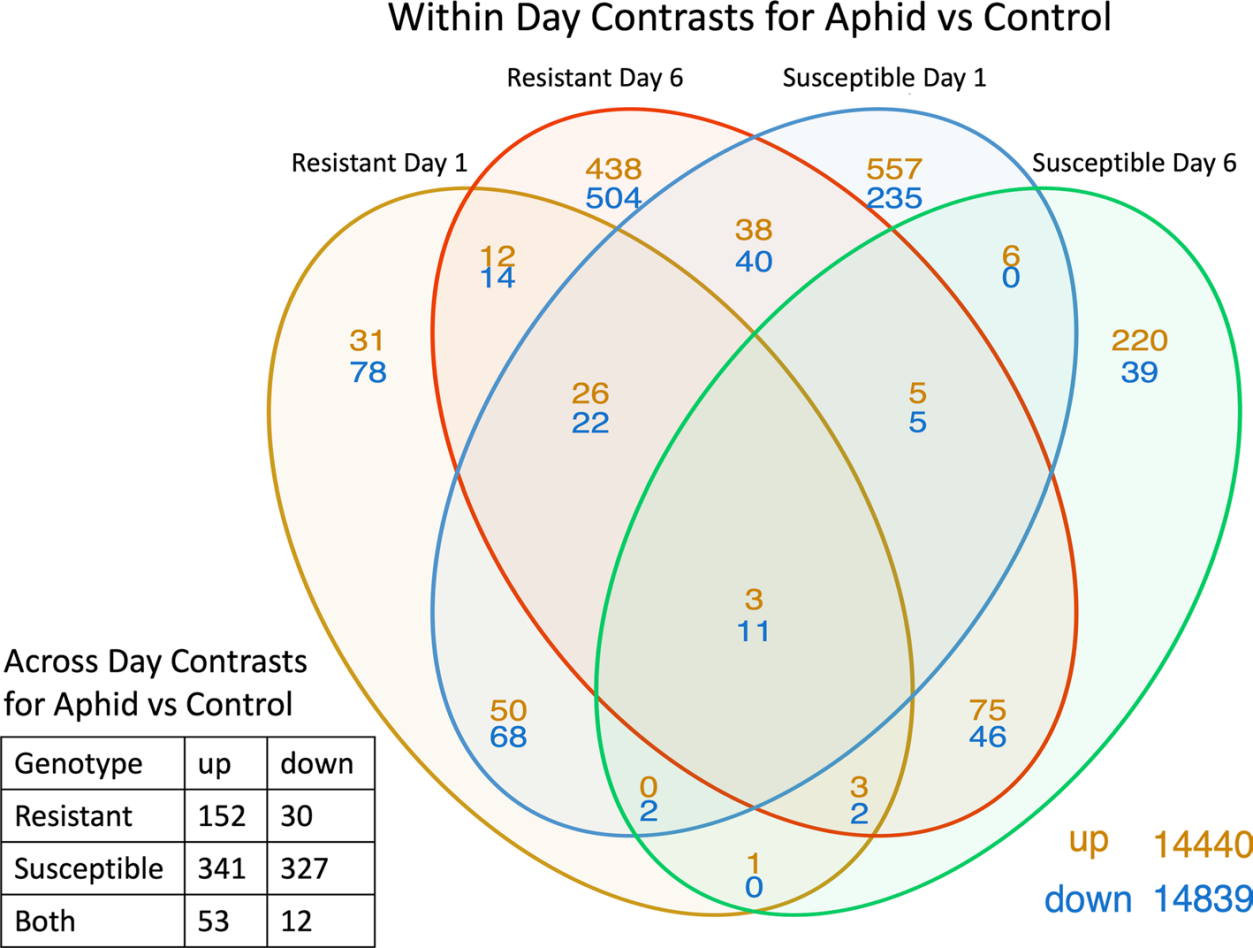
Venn diagram illustration of the number of DEGs up or downregulated by cowpea aphid feeding in susceptible and resistant cowpea genotypes at the two different time timepoints since aphids began feeding, *P* (FDR) < 0.05 and LFC ≥ 0.6 or ≤ -0.6. The table indicates the number of DEG in aphid vs control samples pooled across days and after which development (change in control samples over time) is removed, i.e., the aphid-only effects over time

Immediately after colonization aphids began remodeling cowpea development and signaling, but in a manner dependent on host plant resistance and the duration of feeding. Gene expression changed in both genotypes after one day of feeding, but resistant plants altered expression in only 30% (323 vs 1068) of expressed genes compared to susceptible plants (Fig. 1). With this set of genes, the resistant cowpea line uniquely over-expressed 481 genes and downregulated expression of 596 genes, with most expression differences occurring at the six-day feeding time point (Fig. 1). Susceptible plants initially responded with greater perception of aphid feeding (1068 DEGs after one day of feeding, of which 792 were unique) but this response declined over time with resistant plants differentially expressing 3 × the number of genes (1244) after 6 days of exposure to aphid feeding (susceptible plants showed changes in 418 genes at day six). These patterns highlight the inducible and temporal nature of resistance in cowpea. They are also consistent with aphid performance and visual changes in plant phenotype under aphid attack (Figs. S1, S2), and support performance studies that previously noted antibiosis-based resistance [14].

**Fig. 2:**
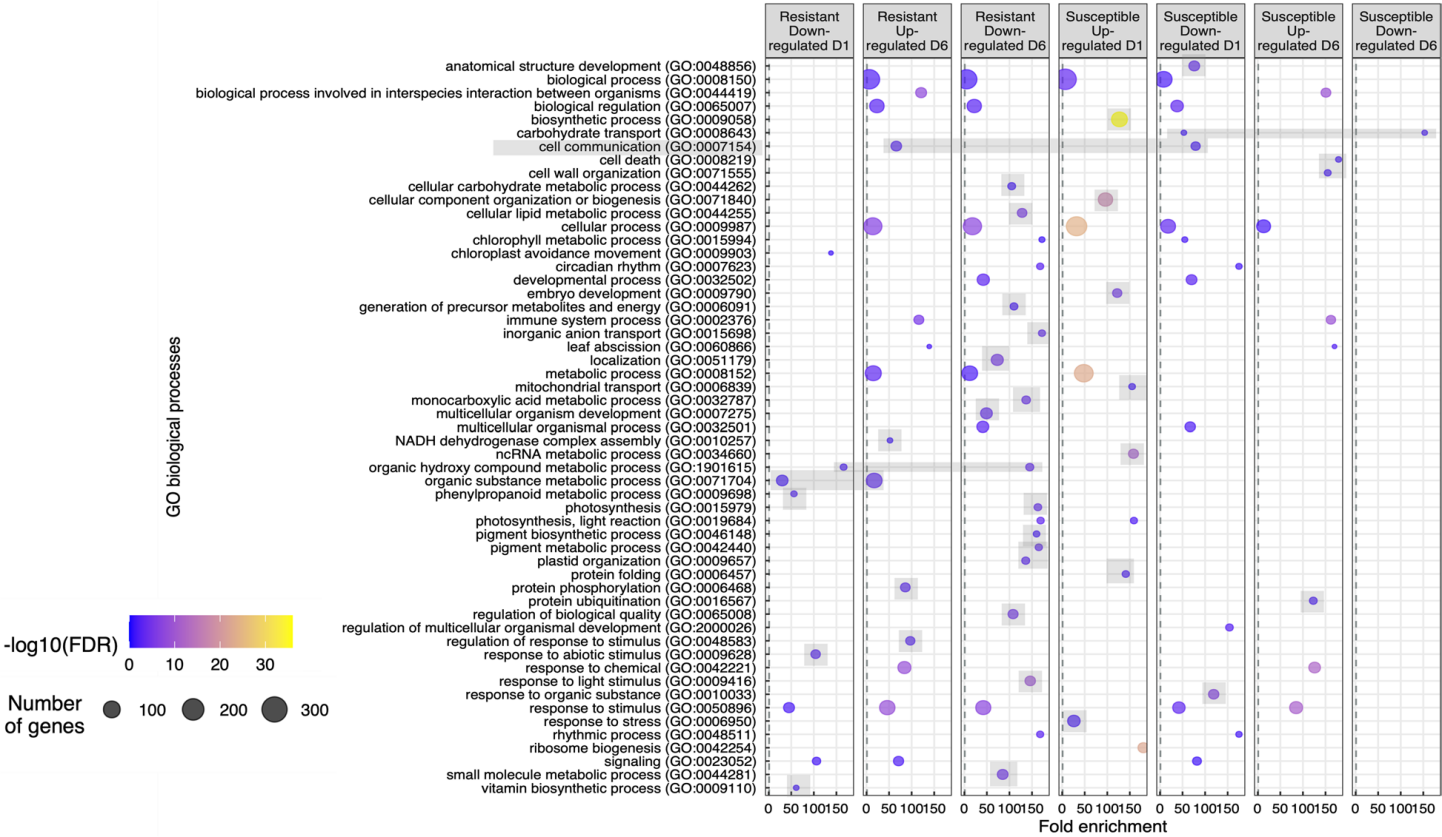
Gene ontology (GO) analyses. Results indicate numerous processes are enriched in a time dependent manner when cowpea aphids feed on resistant and susceptible plants. No pathways were enriched in genes upregulated in resistant plants on day one (not shown). Gray shading indicates processes uniquely expressed within each genotype

Clusters of genes belonging to distinct gene ontologies (GO) highlight key processes that change in response to initial aphid attack and over chronic aphid feeding (Fig. 2). In resistant plants, aphid attack uniquely suppressed phenylpropanoid metabolism (GO: 0009698) and genes responsive to abiotic stress (GO: 0009628), among other vitamin biosynthetic processes (GO: 0009110). Prolonged feeding on resistant plants increased expression in genes related to protein phosphorylation (GO: 0006468) and stimulus response (GO: 00048583) but suppressed processes related to lipids, carbohydrates, and photosynthesis. In contrast, following initial aphid attack, susceptible plants uniquely increased expression of genes in pathways related to stress response, protein folding, noncoding RNA, energy and embryonic processes. But after prolonged feeding for six days, only genes in pathways related to cell death, cell wall production, and ubiquitination were uniquely upregulated. These patterns highlight feeding-induced resistance may depend on kinase dependent signal transduction that attenuates growth in favor of less suitable nutrient status compared to susceptible plants.

Without aphid feeding, resistant plants differed from susceptible plants in constitutive expression of genes related to cell function and secondary metabolite synthesis (Fig. S3). Specifically, resistant plants maintained lower expression in genes related to the cell cycle (GO:0007049, GO:0000278), redox homeostasis (GO:0045454), and tetrapyrrole metabolism (GO:0033013). Susceptible plants uniquely regulated (by fivefold more than resistant plants) processes involved in suppression of N and C metabolism (GO:0019676, GO:0015977), plant growth (GO:0051301, GO:0061640, GO:0120252, GO:0046907, GO:0009657), enhancement of cell turnover (GO:008,219, GO:0012501, GO:0044248), secondary metabolite processes (GO:0019748) specifically flavonoid metabolic processes (GO:0009812), glycosylation (GO:0070085, GO:0006486), and signaling (GO:0023052). These patterns highlight a role for resource acquisition and mobilization in determining the susceptibility to aphid attack. When leaf development is accounted for, but aphids remain on plants, resistant plants show enrichment in very few processes that regulate environmental sensing and signal transduction; however, susceptible plants show enrichment in nearly nine-fold more processes (Fig. S4). These patterns highlight the magnitude of change induced by aphids in susceptible plants.

### Differential gene coexpression identifies gene clusters within treatments

**Fig. 3:**
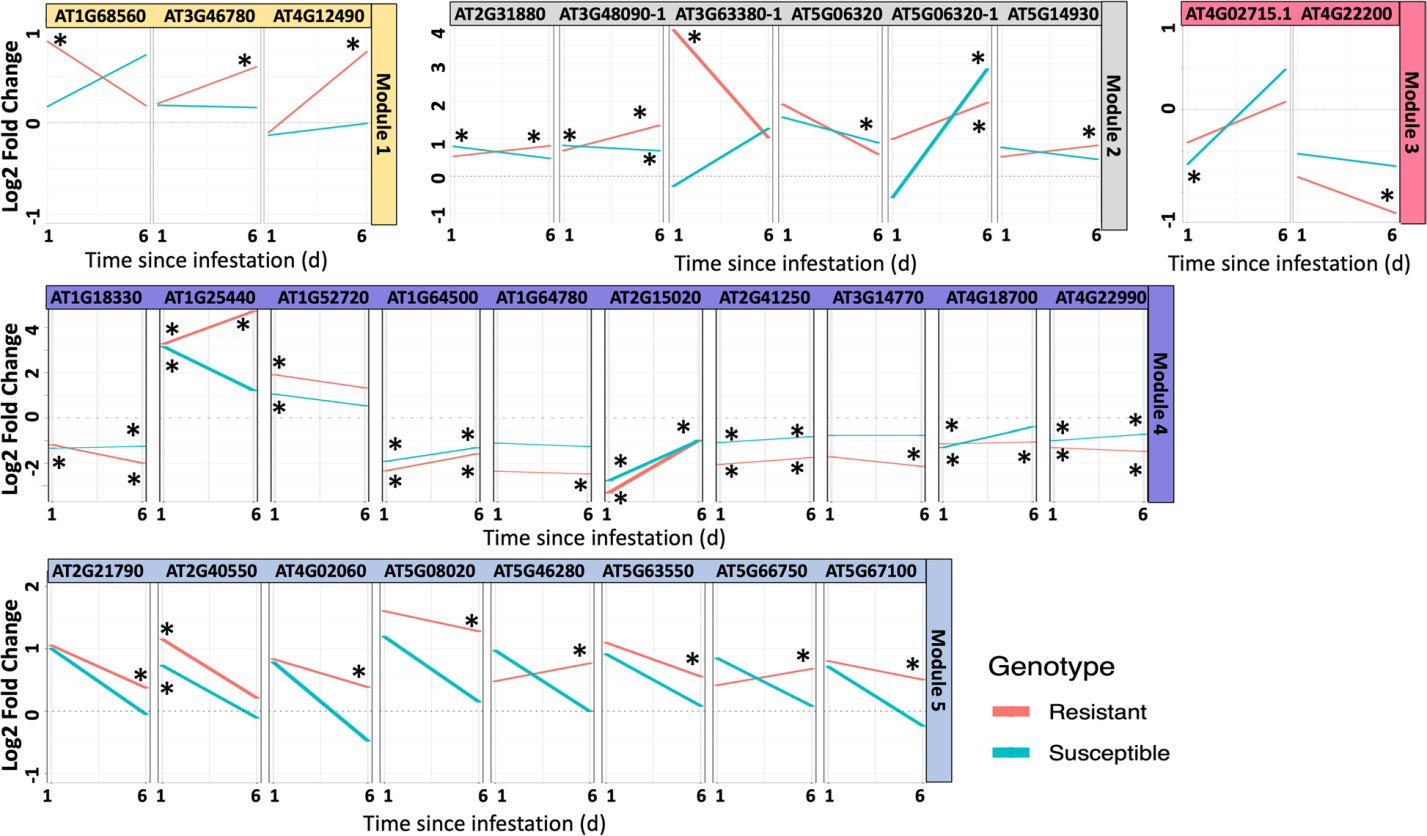
Hub genes within each co-expressed module. Hubs were also differentially expressed in aphid infested *vs.* uninfested plants with respect to genotype and time. Asterisks denote highly expressed |logFC|> 0.6 and significant (FDR < 0.05) genes

Differential coexpression analysis identified clusters (modules: M) of genes that shared expression patterns within treatments but differed between treatments (Fig. 3, Table S1). Seven processes were enriched among three modules. The largest cluster, Module 1, contained an overrepresented sample of photosynthesis and cell wall related genes but these were not differentially enriched (positive or negative NES scores) in aphid or genotype treatments. However, enrichment for all one day feeding treatments indicated that time influenced M1 gene activity the most. The second largest cluster, M2, featured significant overrepresentation of phytohormone and solute transport processes, plus two other processes, response to external stimuli and protein modification, that showed a trend of overrepresentation (at *P* = 0.055). All M2 genes were positively enriched by aphid feeding and expressed in susceptible genotypes; however, across all treatment combinations there were no treatment-specific patterns and again time influenced module activity the most. Of note, three of the six genes in M2 are related to salicylic acid signaling, indicating some involvement of this pathway in resistance. The smallest cluster, M5, featured significant overrepresentation of cell cycle, nucleotide metabolism, and chromatin organization, with enrichment evident due to aphid feeding and in susceptible plants. There was a shift in enrichment of gene sets across all treatments where only resistant genotypes (with Aphid or No Aphid) at day six of aphid feeding showed negative NES scores, indicating downregulated genes. The remaining two modules (M3 and M4) did not contain any overrepresented processes but showed different patterns of gene set enrichment with positive NES scores in control (no aphid) and resistant plants, and only time-dependent enrichment when all treatments were compared.

Within each module, genes with a high degree of connectivity to other similarly co-expressed genes were identified as hubs, and some were also differentially expressed relative to aphid infestation (Fig. 3). Select hub genes from M1 and M5 were overexpressed in resistant plants. M2 hub genes showed variable expression relative to the resistance phenotype whereas hub genes in M3 and M4 were largely suppressed by aphid feeding (Fig. 3). Across all modules, solute transport, RNA remodeling, and response to external stimuli were the most common gene categories identified as hubs. These patterns in coexpression identify candidate regulatory genes and their networks that may enable the resistance phenotype.

### Aphid feeding upregulates DEGs in regions of interest (ROI) in resistant cowpea

The two lines were found to be highly homologous with only 435/49,446 SNPs differing between genotypes. These 435 SNPs are clustered to the aphid resistant QTLs previously identified [10]. The major QTL (7.1) was originally identified on the same contig (337) between markers 1_0912 and 1_0391. The minor QTL (1.1) was identified on two different contigs (407 and 674) between markers 1_0357 and 1_0312. When those markers are applied to the new cowpea genome, the major QTL aligns to chromosome 2 and the minor QTL to chromosome 5 [10, 26]. These chromosomal locations align with the clustering of the 435 SNPs. Of the 435 SNPs, 318 were located on chromosome 2 (Vu02), and 102 were located on chromosome 5 (Vu05).

The source of the aphid resistance is most likely present in these two regions on chromosomes Vu02 and Vu05. By searching the boundaries of these two regions against the publicly available genome on Phytozome, over 400 genes were identified. Of these genes, 305 genes were located on Vu02 and another 111 were located on Vu05. Searching these genes against the DEGs identified in the RNAseq analysis found 180 DEGs on Vu02 and 55 DEGs on Vu05. The expression patterns of both lines of cowpea showed variation in upregulated DEGs. On chromosome Vu02, 24/180 DEGs were constitutively upregulated (baseline resistant) or induced upon aphid feeding in resistant plants after six days. On chromosome Vu05, 3/55 were upregulated in either baseline resistant line samples or samples from resistant plants fed on for six days. All three were only upregulated in resistant cowpea (Table 1).

**Table 1:** Upregulated differentially expressed genes in regions of interest in resistant and susceptible cowpea

Chromosome	Chromosome location	LocusName	Arabidopsis ID	Rice ID	General description	Cowpea line	Contrast	LFC
Vu02	24,041,975–24,044,215	Vigun02g086000	NA	LOC_Os07g30330	cytokinin-O-glucosyltransferase 2, putative, expressed	Resistant	Baseline resistance	0.71
Vu02	24,527,133–24,529,115	Vigun02g090300	AT5G46790	LOC_Os10g42280	cyclase/dehydrase family protein, putative, expressed/REGULATORY COMPONENTS OF ABA RECEPTOR 12	Resistant	Baseline resistance	0.74
Vu02	24,718,128–24,719,022	Vigun02g092000	AT4G17920	NA	ARABIDOPSIS TÃ^3^XICOS EN LEVADURA 29, RING/U-box superfamily protein	Resistant	Baseline resistance	3.60
Vu02	24,893,932–24,895,648	Vigun02g093900	AT1G32260	LOC_Os02g03560	expressed protein/envelope glycoprotein	Resistant	Baseline resistance	0.65
Vu02	25,427,691–25,430,592	Vigun02g099000	AT1G32100	LOC_Os12g16410	isoflavone reductase, putative, expressed/PINORESINOL REDUCTASE 1	Resistant	Baseline resistance	1.63
Vu02	25,434,966–25,437,102	Vigun02g099100	AT1G32100	LOC_Os12g16410	isoflavone reductase, putative, expressed/PINORESINOL REDUCTASE 1	Resistant	Baseline resistance	3.04
Vu02	25,539,416–25,544,157	Vigun02g100400	NA	LOC_Os05g31530	disease resistance protein RGA4, putative, expressed	Resistant	Baseline resistance	3.54
Vu02	25,555,264–25,560,603	Vigun02g100600	NA	LOC_Os05g31530	disease resistance protein RGA4, putative, expressed	Resistant	Baseline resistance	6.62
Vu02	25,566,413–25,573,986	Vigun02g100700	NA	LOC_Os05g31530	disease resistance protein RGA4, putative, expressed	Resistant	Baseline resistance	9.10
Vu02	25,601,805–25,609,187	Vigun02g101300	NA	LOC_Os05g31530	disease resistance protein RGA4, putative, expressed	Resistant	Baseline resistance	1.32
Vu02	25,636,494–25,641,136	Vigun02g101600	NA	LOC_Os05g31530	disease resistance protein RGA4, putative, expressed	Resistant	Baseline resistance	1.31
Vu02	25,647,631–25,651,399	Vigun02g101800	AT5G45400	LOC_Os05g02040	RPA1C—Putative single-stranded DNA binding complex subunit 1, expressed/Replication protein A 70 kDa DNA-binding subunit C	Resistant	Baseline resistance	0.64
Vu02	25,717,359–25,721,986	Vigun02g102400	NA	LOC_Os05g31530	disease resistance protein RGA4, putative, expressed	Resistant	Baseline resistance	3.82
Vu02	25,733,262–25,737,804	Vigun02g102500	NA	LOC_Os05g31530	disease resistance protein RGA4, putative, expressed	Resistant	Baseline resistance	1.19
Vu02	25,744,177–25,748,760	Vigun02g102600	NA	LOC_Os05g31530	disease resistance protein RGA4, putative, expressed	Resistant	Baseline resistance	3.07
Vu02	25,866,019–25,878,677	Vigun02g104200	AT3G23640	LOC_Os07g23880	glycosyl hydrolase, family 31, putative, expressed/HETEROGLYCAN GLUCOSIDASE 1	Resistant	AphidResD6	0.73
Vu02	25,995,838–26,000,843	Vigun02g105800	AT5G45275	LOC_Os11g01590	nodulin, putative, expressed/Major facilitator superfamily protein, solute transport	Resistant	Baseline resistance	0.63
Vu02	26,005,896–26,009,931	Vigun02g105900	AT3G47570	LOC_Os01g05980	receptor kinase, putative, expressed/Leucine-rich repeat protein kinase family protein	Resistant	Baseline resistance	1.43
Vu02	26,031,026–26,037,601	Vigun02g106300	AT4G19420	LOC_Os02g47400	pectinacetylesterase domain containing protein, expressed/PECTIN ACETYLESTERASE 8	Resistant	Baseline resistance	1.17
Vu02	26,088,276–26,093,541	Vigun02g107000	AT1G31650	LOC_Os02g47420	ATROPGEF7/ROPGEF7, putative, expressed/ROP (RHO OF PLANTS) GUANINE NUCLEOTIDE EXCHANGE FACTOR 14	Resistant	AphidResD6	0.83
Vu02	26,158,748–26,165,517	Vigun02g107600	AT4G19380	NA	Long-chain fatty alcohol dehydrogenase family protein	Resistant	AphidResD6	0.75
Vu02	26,371,444–26,373,970	Vigun02g109800	AT4G19170	LOC_Os02g47510	9-cis-epoxycarotenoid dioxygenase 1, chloroplast precursor, putative, expressed/CAROTENOID CLEAVAGE DIOXYGENASE 4	Resistant	Baseline resistance	0.61
Vu02	26,389,324–26,394,122	Vigun02g110100	AT3G45890	LOC_Os04g22360	DUF647 domain containing protein, putative, expressed/Ribonuclease P protein subunit P38-like protein	Resistant	AphidResD6	0.64
Vu02	26,414,667–26,416,541	Vigun02g110300	AT1G14130	LOC_Os04g39980	gibberellin 20 oxidase 2, putative, expressed/DIOXYGENASE FOR AUXIN OXIDATION 1	Resistant	Baseline resistance	1.61
Vu05	4,416,033–4,419,347	Vigun05g051600	AT5G48370	LOC_Os09g34190	acyl-coenzyme A thioesterase 10, mitochondrial precursor, putative, expressed/Thioesterase/thiol ester dehydrase-isomerase superfamily protein	Resistant	Baseline resistance	1.09
Vu05	4,505,968–4,507,948	Vigun05g052700	AT3G60200	LOC_Os02g46420	transposon protein, putative, unclassified, expressed/Uncharacterized protein	Resistant	AphidResD6	1.65
Vu05	4,577,021–4,580,973	Vigun05g053400	AT3G03305	LOC_Os01g66920	Ser/Thr protein phosphatase family protein, putative, expressed/Calcineurin-like metallo-phosphoesterase superfamily protein	Resistant	AphidResD6	0.73
Vu02	24,637,099–24,637,783	Vigun02g091400	AT1G32690	LOC_Os02g46620	expressed protein/DUF740 family protein	Susceptible	AphidSuscD6	0.72
Vu02	24,707,273–24,709,788	Vigun02g091900	AT5G17600	LOC_Os04g50100	RING-H2 finger protein ATL5G, putative, expressed/RING/U-box superfamily protein	Susceptible	AphidSuscD6	3.11
Vu02	25,324,008–25,326,837	Vigun02g097700	AT3G07040	LOC_Os08g32880	disease resistance protein RPM1, putative, expressed/NB-ARC domain-containing disease resistance protein,RPM1	Susceptible	AphidSuscD6	1.20
Vu02	26,773,059–26,776,235	Vigun02g114000	NA	NA	NA	Susceptible	AphidSuscD6	3.71
Vu02	25,779,331–25,781,605	Vigun02g103100	AT3G49010	LOC_Os06g02510	ribosomal protein L13, putative, expressed/60S ribosomal protein L13	Susceptible	AphidSuscD1	0.88
Vu02	26,077,542–26,082,129	Vigun02g106900	NA	NA	NA	Susceptible	AphidSuscD1	3.11
Vu05	4,141,516–4,146,610	Vigun05g048200	AT4G16830	LOC_Os01g52390	plasminogen activator inhibitor 1 RNA-binding protein, putative, expressed/Hyaluronan / mRNA binding family	Susceptible	AphidSuscD1	0.70

Of the 24 DEGs upregulated in Vu02 resistant cowpea, 20 were identified in baseline resistant plant samples and the other four were identified in resistant plants fed on by aphids for six days (Table 1). This difference before the presence of aphids indicates that the resistance mechanism in CB77 plants is at least partially constitutive.

Interestingly, 8/20 upregulated DEGs in baseline resistant plant samples were identified as the same gene, disease resistance protein RGA4 (Vigun02g100400, Vigun02g100600, Vigun02g100700, Vigun02g101300, Vigun02g101600, Vigun02g102400, Vigun02g102500, Vigun02g102600). Multiple isoflavone reductases (Vigun02g099000, Vigun02g099100) were also identified among the upregulated DEGs baseline resistant plant samples as well as a leucine rich repeat protein kinase (Vigun02g105900).

Two of the three DEGs upregulated on Vu05 were identified in the six-day aphid feeding resistant plant samples and the other DEG was identified in samples from baseline resistant plants (Table 1). The upregulated DEG from baseline resistant plant samples was identified as a thioesterase (Vigun05g051600). One of the two DEGs upregulated in samples from resistant plants fed on for six days was uncharacterized (Vigun05g052700) and the other was identified as a phosphatase (Vigun05g053400).

The regions on Vu02 and Vu05 contain the majority of genetic differences between the two lines and analyzing this region in the CB46 could partially identify DEGs involved in the damage symptoms observed during cowpea aphid infestation [14]. In the CB46 there were very few upregulated DEGs in these two regions (Table 1). On Vu02, only two DEGs were upregulated in susceptible cowpea after one day of feeding but four were upregulated after 6d sustained feeding. There were even fewer genes upregulated on Vu05 with only one, an mRNA binding protein (Vigun05g048200) Interestingly, one of the DEGs upregulated was identified as a CNL, disease resistance protein RPM1 (Vigun02g097700). Upregulation in CB46means RPM1 is not effective in resistance against cowpea aphid infestation but could be providing resistance to other stressors. Two of the seven total DEGs that were upregulated had no annotation or were unknown (Vigun02g114000 and Vigun02g106900).

### Aphid feeding downregulates DEGs in regions of interest (ROI) in resistant cowpea

We also detected downregulated DEGs located in ROIs on Vu02 and Vu05 (Table 2). On Vu02 only 12/180 DEGs were suppressed by aphids, with 7/12 downregulated only in resistant cowpea. Of these genes, five were identified in the resistant plant samples subjected to six days of aphid feeding and the other two were identified in plant samples fed on for one day. Four of seven genes were unknown or uncharacterized. On Vu05 7/55 DEGs were downregulated by aphids and whereas four were only downregulated in resistant cowpea (Table 2). Three of the DEGs were identified in resistant plant samples fed on by aphids for six days and the other one was identified on resistant plant samples fed on for one day. One of the DEGs downregulated in resistant plant samples fed on for six days was identified as a leucine rich repeat protein kinase (Vigun05g048800). The downregulation of this leucine rich repeat protein kinase in the resistant NIL in response to aphid infestation indicates Vigun05g048800 could be acting in a regulatory role.

**Table 2:** Downregulated differentially expressed genes in regions of interest in resistant and susceptible cowpea

Chromosome	Chromosome location	Locus Name	Arabidopsis ID	Rice ID	General description	Cowpea line	Contrast	LFC
Vu02	24,527,133–24,529,115	Vigun02g090300	AT5G46790	LOC_Os10g42280	cyclase/dehydrase family protein, putative, expressed/REGULATORY COMPONENTS OF ABA RECEPTOR 12	Resistant	AphidResD6	-1.09
Vu02	25,088,659–25,091,739	Vigun02g095300	AT1G32170	LOC_Os02g03550	glycosyl hydrolases family 16, putative, expressed/XYLOGLUCAN ENDOTRANSGLUCOSYLASE/HYDROLASE 30	Resistant	AphidResD1	-0.96
Vu02	25,281,475–25,283,889	Vigun02g097000	AT1G31920	LOC_Os02g46980	pentatricopeptide, putative, expressed/Pentatricopeptide repeat-containing protein	Resistant	AphidResD6	-1.27
Vu02	25,295,229–25,295,454	Vigun02g097200	NA	NA	NA	Resistant	AphidResD1	-3.91
Vu02	25,986,925–25,987,906	Vigun02g105500	AT2G41342	NA	Uncharacterized protein	Resistant	AphidResD6	-1.97
Vu02	26,014,360–26,015,720	Vigun02g106000	NA	NA	NA	Resistant	AphidResD6	-0.96
Vu02	26,603,320–26,607,301	Vigun02g111900	NA	NA	NA	Resistant	AphidResD6	-0.74
Vu05	3,884,709–3,887,992	Vigun05g046400	AT3G60390	LOC_Os10g41230	homeobox associated leucine zipper, putative, expressed/Homeobox-leucine zipper protein HAT3	Resistant	AphidResD6	-1.44
Vu05	4,194,856–4,199,405	Vigun05g048800	AT1G35710	NA	kinase family with leucine-rich repeat domain-containing protein	Resistant	AphidResD6	-2.79
Vu05	4,360,865–4,362,890	Vigun05g050900	AT2G44740	LOC_Os10g41430	cyclin, putative, expressed/CYCLIN P4;1	Resistant	AphidResD6	-1.68
Vu05	4,416,033–4,419,347	Vigun05g051600	AT5G48370	LOC_Os09g34190	acyl-coenzyme A thioesterase 10, mitochondrial precursor, putative, expressed/Thioesterase/thiol ester dehydrase-isomerase superfamily protein	Resistant	AphidResD1	-0.68
Vu02	24,637,099–24,637,783	Vigun02g091400	AT1G32690	LOC_Os02g46620	expressed protein/DUF740 family protein	Susceptible	AphidSuscD1	-0.63
Vu02	24,760,762–24,763,564	Vigun02g092400	AT4G17790	LOC_Os02g46660	SNARE associated Golgi protein, putative, expressed	Susceptible	AphidSuscD1	-0.64
Vu02	25,651,668–25,656,208	Vigun02g101900	AT4G19140	LOC_Os04g51166	expressed protein/exopolysaccharide production negative regulator	Susceptible	AphidSuscD1	-0.66
Vu02	26,014,360–26,015,720	Vigun02g106000	NA	NA	NA	Susceptible	AphidSuscD1	-1.01
Vu02	26,466,136–26,473,697	Vigun02g110900	AT3G51520	LOC_Os02g48350	diacylglycerol O-acyltransferase, putative, expressed/DIACYLGLYCEROL ACYLTRANSFERASE 2	Susceptible	AphidSuscD1	-0.81
Vu05	4,360,865–4,362,890	Vigun05g050900	AT2G44740	LOC_Os10g41430	cyclin, putative, expressed/cyclin p4;1	Susceptible	AphidSuscD1	-1.20
Vu05	4,487,115–4,489,745	Vigun05g052500	AT1G44750	LOC_Os02g46380	purine permease, putative, expressed/purine permease 11	Susceptible	AphidSuscD1	-0.63
Vu05	4,505,968–4,507,948	Vigun05g052700	AT3G60200	LOC_Os02g46420	transposon protein, putative, unclassified, expressed/Uncharacterized protein	Susceptible	AphidSuscD1	-1.18

There was a similar number of downregulated DEGs in regions of interest on Vu02 and Vu05 in CB46 compared to those that were upregulated (Table 2). On Vu02, five genes were downregulated uniquely in susceptible cowpea whereas on Vu05 three DEGs were downregulated uniquely in susceptible cowpea. The suppression of any one or multiple genes in these ROI may play a role in aphid resistance likely through a yet unresolved interaction network.

## Discussion

Our results demonstrate that feeding by the cowpea aphid globally remodels the transcriptome of cowpea, but that how this occurs depends on both the duration of feeding and host-plant resistance. Constitutive expression profiles of the resistant NIL suggest that cowpea aphid resistance in this genotype partially involves a finely-tuned resource management strategy that ultimately reduces damage (e.g., chlorosis) and delays cell turnover, while impeding aphid performance. Constitutive expression of aphid resistance has been identified in several other plant species [27–29]. Previously, it was determined that the resistance mechanism present in CB77 had antibiosis and antixenosis components, with antixenosis being constitutively present [10, 14]. Other aphid resistance genes identified are linked to both antibiosis and antixenosis components, such as the *Rag* genes in soybean (*Glycine max*) that confer resistance to the soybean aphid (*Aphis glycines*). Of the12 *Rag* genes identified, *Rag1* and *Rag5* are two *R* genes that mediate antibiosis and antixenosis components [30–33]. Additionally, *Rag1* and *Rag5*, both confer constitutive baseline resistance similar to the resistance identified in cowpea in this study [29, 34]. But our time course experiment revealed that constitutive defenses are not the only resistance mechanism operating in CB77; DEG profiles resulting from different contrasts in this study uncovered multiple changes in key processes following aphid infestation (Figs. 2, 3). While some of these processes are present constitutively, there are also distinct gene expression changes in the two regions of interest (ROI) where differences between the susceptible and resistant lines are concentrated (Tables 1, 2). These data suggest both constitutive and inducible mechanisms of resistance are operating in NIL CB77.

Our time course damage experiment provided insight into the mechanisms underlying constitutive and induced resistance in CB77. We found few DEGs in the resistant plants at one day post infestation (Fig. 1), suggesting constitutive and not inducible expression plays a role in the behavioral avoidance and feeding difficulties observed in the first 24 h after aphid contact with plants [14, 29]. Soybean aphids feeding on *Rag1* resistant soybean also did not induce differential gene expression during the first day of aphid infestation. It is possible that reduced DEGs in the resistant cowpea line after one day of aphid exposure may be due to reductions in aphid feeding. Our prior EPG analysis revealed that aphids struggled to feed on resistant plants during the first eight hours of contact. Less feeding may lead to reduced exposure to aphid salivary components (especially via phloem contact), which could result in reduced differential expression. This conclusion is supported by the presence of substantial numbers of DEGs in resistant plant samples taken after six days of aphid feeding relative to those from susceptible plants undergoing the same aphid attack (Fig. 1). In contrast, for soybean, there was only one DEG detected in longer infestation times [27, 29]. In both cowpea and soybean, genetic differences between the resistant and susceptible lines determine inducibility of defenses, but for cowpea, inducible defense seems to be a more substantial component of the overall resistance phenotype.

Besides the lack of DEGs present in resistant plants fed on by aphids for one day, there were notable differences in responses to aphid infestation in other resistance x time point combinations. In susceptible cowpea fed on by aphids for one day, there were 552 unique DEGs upregulated (Fig. 1). Compared to all other resistance x feeding duration treatments, the susceptible plants after one day of feeding had a high representation of DEGs involved in biosynthetic processes (GO:0009058), cellular processes (GO:0009987), and metabolic processes (GO:0008152). Concentration of DEG activity around cellular and synthesis processes could be the result of plants activating processes that support defense responses and/or aphid-mediated manipulation to generate a local sink of plant resources for ingestion [15]. The fact that aphids readily exploit susceptible plants suggests the latter possibility.

Throughout the aphid infestation, specific clusters of similar DEGs varied depending on plant genotype (Figs. 2, 3). Differentially expressed gene clusters have been reported in studies of other aphid-plant systems [28] and provide insight into what genes and pathways are responsible for the resistance being conferred. In the absence of a single *R* gene conferring resistance, it is more likely a complex response with a number of genes and pathways being involved. For example, in sorghum (*Sorghum bicolor*), a sugarcane aphid resistant line had more upregulation of protein and lipid binding controlling genes, cellular catabolic processes, as well as increased transcriptional initiation after sugarcane aphid infestation compared to the susceptible line [28]. In cowpea (Fig. 3) the first gene cluster (M1) representing photosynthesis and cell wall related genes, and fifth gene cluster (M5) representing cell cycle, nucleotide metabolism, and chromatin organization were more differentially expressed in the resistant line compared to the susceptible. Interestingly, similar types of gene clusters including photosynthesis related genes and nucleotide binding related genes were upregulated in sugarcane aphid resistant sorghum in the undamaged (0 h) time point [28]. In cowpea, the majority of the genes in these two clusters were upregulated in the resistant plants at the 6-day time point (Fig. 3). These differences indicate that genes central to global expression patterns (i.e. hubs) that function similarly are differentially induced by biotic stress depending on genotype, in addition to possible variation in timing of gene hub regulation across species.

Most of the differences between the genotypes in our study are concentrated in two major regions of interest, one on chromosome Vu02 (major QTL) and another on chromosome Vu05 (minor QTL). Differential expression of genes in these areas likely underlies the divergent aphid resistance and damage response phenotypes exhibited by each line, as well as associated remodeling of transcriptomes in response to aphid damage. In both ROIs, there were fewer DEGs detected in susceptible CB46 than the resistant NIL; with only 7 upregulated DEGs and 8 downregulated DEGs (Tables 1, 2). Of the upregulated DEGs, three were uncharacterized or unknown and one was identified as the disease resistance protein RPM1 (Vigun02g097700). RPM1 is a CNL that provides resistance to *Pseudomonas syringae* [35]. The upregulation of *RPM1* only in the susceptible line indicates it does not provide resistance to the cowpea aphid. In the downregulated DEGs there was one protein uncharacterized or classified as unknown (Vigun02g106000; Table 2).

Gene expression differences in the ROI for the resistant NIL, also revealed genes that may be integral to the aphid response. One differentially expressed gene of note is *RGA4,* located in the region of interest on chromosome Vu02 (Table 1). RGA4 is a CNL first identified in rice (*Oryza sativa*) as a source of resistance to the fungal pathogen *Magnaporthe oryzae* [36]. In *O. sativa*, RGA4 works with another CNL, RGA5 to provide resistance to *M. oryzae*. RGA4 and RGA5 form a heterodimer with RGA5 acting as a repressor to RGA4 and as a receptor for avirulence (AVR) proteins. Perception of an AVR protein by RGA5 leads to RGA4 triggered cell death [37, 38]. There were eight cowpea DEGs upregulated in the resistant NIL that were identified as an RGA4 homolog (Vigun02g100400, Vigun02g100600, Vigun02g100700, Vigun02g101300, Vigun02g101600, Vigun02g102400, Vigun02g102500, Vigun02g102600). While there was no identification of an *RGA5* homolog in any of the contrasts, BLAST searches indicate there are RGA5 homologs encoded in the cowpea genome. This could be due to a lack of difference in *RGA5* expression after cowpea aphid infestation. It is also possible that *RGA4* regulation differs among plant species. For example, in our study, *RGA4* was constitutively expressed in the resistant cowpea NIL (Table 1) but constitutive expression of *RGA4* in rice leads to cell death [38]. The constitutive expression of *RGA4* is in line with the initial aphid deterrence previously identified in the resistant NIL.

Another top candidate for the resistance present in CB77 is the leucine-rich repeat protein kinase, Vigun02g105900, which was expressed in undamaged CB77 plants (Table 1). The protein encoded by this gene contains two transmembrane helices and is likely a receptor like kinase (RLK). RLKs are a subset of PRRs and function by triggering an immune response following recognition of molecular patterns associated with pathogens or pests [39, 40]. The constitutive expression of this RLK could lead to a quicker initiation of defense responses upon infestation.

The DEGs associated with the minor QTL region of intereston chromosome Vu05 may also play a role in cowpea aphid resistance [10, 26]. One of the three upregulated DEGs in this region of interest was identified as an uncharacterized protein, Vigun05g052700 (Table 1). This uncharacterized DEG was upregulated in resistant plants fed on by aphids for six days. Similarly, Vigun05g053400, a phosphatase, was also upregulated in resistant plants after six days of aphid feeding (Table 1). Phosphatases are known regulators of plant immunity and the upregulation of Vigun05g053400 may be involved in inducible resistance in line CB77 [41]. The third DEG, Vigun05g051600, a thioesterase, was upregulated in undamaged resistant plants. Previously, thioesterases in plants have been found to produce secondary metabolites that have insecticidal activity [42, 43].

Downregulated genes located in regions of interest on Vu02 and Vu05 also show genotype-specific patterns of expression. There are several Vu02-located DEGs that are uncharacterized or lack annotation that were downregulated in resistant plants fed on by aphids for both one day and six days (Table 2) (Vigun02g097200, Vigun02g105500, Vigun02g106000, Vigun02g111900). More information on possible identity was only available for Vigun02g111900. Through identification of the soybean homolog on Phytozome, Vigun02g111900 was identified as a *MONOSACCHARIDE-SENSING PROTEIN 2*. This protein could be involved in perception of sugars acting as signaling molecules in an immune response, or sugars used as substrates in direct defenses (e.g., lignification or flavonoid synthesis) [44]. On Vu05, a leucine-rich repeat protein kinase with a transmembrane helix (Vigun05g048800), most likely an RLK, was downregulated in resistant plants aftersix days of aphid feeding(Table 2). Vigun05g048800 may be acting as a negative regulator of immunity to aphids, with down-regulation increasing plant defense responses.

The RGA4 homologs and Vigun02g105900, as likely NLRs, are top candidate cowpea aphid resistance genes. However, all DEGs in these regions, not just NLRs, need to be considered as potential sources of resistance. In soybean, resistance to the soybean cyst nematode (*Heterodera glycines*) was identified and initially believed to be from LRR kinase genes present in the QTLs *rhg1* and *Rhg4*. However, resistance present in these QTLs was independent of LRR kinases [45, 46]. Instead of LRR kinase genes, resistance was the result of 10 tandem copies of a 31-kb segment with three genes: an amino acid transporter, an N-ethylmaleimide-sensitive factor attachment protein, and a wound-inducible protein, each contributing to resistance [47]. The susceptible soybean cultivar Williams 82 contains only a single copy of *rhg1-b* while the resistant cultivars Peking and Fayette have 3 and 10 copies, respectively [47]. The complexity of the resistance to soybean cyst nematode in soybean was unexpected and goes beyond the one gene model. Given that resistance in CB77 is linked to two QTLs, this level of complexity could be in play in the genotypes in our study.

## Conclusions

By employing closely related lines with genetic differences concentrated in two regions of interest, our RNAseq uncovered multiple candidate genes with potential roles in cowpea aphid resistance. By including two time points of aphid damage, as well as undamaged plants, we showed divergent transcriptome reprogramming between the two genotypes and provide evidence that resistance in CB77 functions through both constitutive and inducible gene expression. This finding is consistent with our prior studies documenting aphid performance and behavior on the lines employed here. Constitutive antixenosis was detected through behavioral experiments, while antibiosis was detected through no-choice feeding experiments [14]. The results of this study will inform next steps to functionally validate the roles of candidate genes in the aphid resistance phenotype, for instance, by knockout of gene targets using CRISPR/Cas9 technology or transient silencing using RNA interference.

## Methods

### Plants and aphids growth conditions

The cowpea aphids were collected in the summer of 2016 from a field in Riverside, California. The aphids were reared on cowpea line California Blackeye 46 (CB46) in a pesticide free greenhouse or a plant growth chamber (Conviron) at 28 ± 2 °C with a 16:8 light:dark photoperiod.

Both cowpea lines, CB46 and the NIL CB77, were grown in UC Mix 3 soil (agops.ucr.edu/soil/) in 24 oz plastifoam cup and fertilized weekly with MiracleGro (18 − 18 − 21; Stern’s MiracleGro Products). Plants were maintained in a pesticide free plant growth room at 28 ± 2 °C with a 16:8 light:dark photoperiod.

### RNAseq Experimental design

To determine what genes underlie resistance to cowpea aphid, the transcriptional response of *V. unguiculata* lines CB46 and CB77, were surveyed after one day and six days of continuous aphid infestation. These timepoints were selected because preliminary observations revealed aphid performance declined at 7d on resistant plants (Figure S2). Cowpea aphids do not prefer resistant cowpea and feed significantly less on this genotype [14]. The one day feeding time point will capture early signaling during colonization whereas the six-day feeding time point should reflect induced susceptibility and/or resistance. Accordingly, two-week-old plants from both cowpea lines were infested with 20 adult apterous aphids on a single unifoliate leaf and enclosed in a mesh sleeve bag. Control non-infested plants received an empty mesh sleeve bag. Samples were collected at 1 and 6 days after infestation. Each sample consisted of three unifoliate leaves pooled from three plants. Five biological replicates, each consisting of three pooled plants, per line were used. For both plant lines, the number of aphids were counted on the sixth day of feeding before harvest. The cowpea aphids were removed by immersing the leaves in water and gently removing any remaining aphids with a fine-tip paintbrush. Non-infested control plants were treated the same way. Leaves were immediately frozen in liquid nitrogen and stored at -80 °C until RNA extraction.

### RNA Extraction and library preparation

Leaf tissues were ground to a fine powder in liquid nitrogen with a mortar and pestle. Total RNA from leaves was isolated using the NucleoSpin RNA Plant kit (Macherey–Nagel). RNA was quality checked and quantified using a Bioanalyzer RNA Nano Kit (Agilent, Santa Clara, CA, USA). RNA samples were prepared for Illumina sequencing using the NEB Ultra II RNAseq stranded kit (New England Biolabs, Ipswich, MA, USA) with unique dual index adapters following manufacturers recommendations. Individual libraries were pooled based on BioAnalzyer molarities (Agilent, Santa Clara, CA, USA) and sent to UCLA for 2 × 150 bp sequencing on a NovaSeq 6000 (Illumina, San Diego, CA, USA) and demultiplexed with BCL2FASTQ**.**

### Analysis of RNAseq

Raw RNA-Seq reads were assessed for quality using FastQC [48]; and adapter trimmed using BBDuk (sourceforge.net/projects/bbmap/; default settings: from the right with k = 23, mink = 11, hdist = 1). Reads were mapped to the annotated cowpea reference genome (*V. unguiculata* v1.2; Phytozome) using STAR v2.57a [49] Reads ranged from 69–94% alignment but averaged 87% (Table S2), a high rate of alignment given the repetitive nature of the genome [26]. Counts were filtered to remove zero and low count genes using filterByExpr (min.count = 20), and remaining genes were compared for differential gene expression using EdgeR-limma [50]. These genes cluster well within treatments with time driving the greatest separation among samples (Fig. S5). The linear model y ~ 0 + trt was used to compare all treatment combinations. Counts were normalized using voomWithQualityWeights to account for sample heterogeneity among treatment conditions. The raw sequences were deposited in NCBI as BioProject: PRJNA743032.

Genes were determined significant at the adjusted *P* value (FDR) < 0.05 and logFC ≥ 0.6 or ≤ -0.6 (fold change ≥ 1.5 or ≤ -1.5). Genes of interest were extracted from contrasts after multiple testing for each contrast separately (decideTests: method = “separate”, i.e., top Table). Contrasts of treatments and their combinations were made as defined in Table S3. GO analyses were performed following [51] where best hit *Arabidopsis thaliana* IDs were used to identify GO terms in Panther [52]. Categories were separated by up or down-regulated genes per contrast and considered enriched at FDR < 0.05. Enriched families were clustered by semantic similarity in REVIGO [53] using default settings to visualize GO terms among contrasts and reduce redundancy by extracting terms with “dispensability” (d) ≤ 0.1. Most unique terms are highlighted using d ≤ 0.05.

A differential coexpression analysis was performed to assess what gene networks and hubs associate with resistance phenotypes, aphid infestation, and aphid x genotype treatments using Cemitool [54]. Our analysis at the main treatment level (aphid infestation or genotype) was adequately powered n ~ 20 [55]; however, we also included results of aphid x genotype treatments when patterns were consistent. For ease of visualization, cowpea proteins were functionally classified using Mercator4 v3.0 [56] to assign protein functional annotations and perform over-representation analyses in Cemitool. The resulting gene list was compared with classes defined as above treatments. A subset of genes with known orthologues to *A. thaliana,* as defined by best blast hits in the genome annotation file (*V. unguiculata* v1.2) were used alongside known *A. thaliana* gene interactions [57] to construct interaction networks. Default settings were used except that the minimum threshold of genes per module was reduced to 15 because of the data reduction when selecting only orthologous genes.

A subset of the DEGs were further annotated using their matching *A. thaliana* IDs in TAIR and UniProt to identify molecular function [57, 58]. Predicted cowpea protein sequences of others were further annotated using TMHMM V2.0 and NCBI Conserved Domain searches. Select genes of RGA4 (orthologs Vigun02g100700.1 and Vigun02g100600.1), CHiB (Vigun09g278000), and STE11 (Vigun03g278000) were assessed by qRT-PCR (CFX connect Real-Time system, BIO-RAD, with PowerTrack SYBR Green) against the housekeeping gene UBQ11 (Vigun02g198500.1) following [59]. Three biological replicates were used from each treatment combination and compared with uninfested/control samples of each time point using ANOVA with post hoc t-tests (*P* ≤ 0.05) (Table S4, Fig. S6).

## Supplementary Information

**Additional file 1: Figure S1.** Damage induced by cowpea aphids after 7-days feeding on susceptible cowpea results in chlorosis and necrosis near feeding sites and leaf curling. Photos depict week-old plants infested with 15 adult apterous aphids. **Figure S2.** Growth of cowpea aphid population on susceptible (CB46) and resistant (CB77) cowpea. Two-week-old plants were infested with 20 adult apterous aphids on a single unifoliate leaf and enclosed in a mesh sleeve bag for 1 week. The aphid population was counted daily. Data from MacWilliams et al. 2022. Figure S3. Gene ontology (GO) analyses indicated numerous processes enriched in resistant and susceptible plants over time (day 6 vs day 1) that may play a role in constitutive resistance. Grey shading indicates processes uniquely expressed within each genotype. **Figure S4.** Gene ontology (GO) analyses indicated numerous processes are enriched when cowpea aphids feed on susceptible plants, with more processes upregulated than downregulated after correcting for leaf development. Resistant plants show enrichment in only 20% (6/30) of the total pathways enriched in susceptible plants when aphids feed. **Figure S5.** MDS plots for RNAseq show separation of time points. Overall, each treatment clusters together with the largest separation explained by time. **Figure S6.** qRT-PCR validation of select genes differentially expressed in RNAseq data relative to susceptible (CB46) uninfested plants. Genes consistently upregulated across time in resistant cowpea (CB77; A:RGA4-600, B:RGA4-700) and genes differentially expressed among treatments (C:CHiB, D:STE11) showed expression trends in concordance with RNAseq data (*) relative to susceptible control tissues at the collection time point. Primers for select genes are listed (E) and the fit of all expression data to count data showed high correlation (F: R2=0.79). 

**Additional file 2: Table S1.** Differential coexpression analysis identified clusters (modules: M) of genes that shared expression patterns within treatments but differed between treatments (see Figure 3). **Table S2.** Alignment statistics for raw reads. **Table S3.** Contrasts of treatments and associated DEGs at high and low LFC thresholds. **Table S4.** qRT-PCR validation of select genes differentially expressed in RNAseq data relative to susceptible (CB46)  uninfested plants. Genes consistently upregulated across time in resistant cowpea (CB77; A:RGA4-600, B:RGA4-700) and genes differentially expressed among treatments (C:CHiB, D:STE11) showed expression trends in concordance with RNAseq data (*) relative to susceptible control tissues at the collection time point. Primers for select genes are listed (E) and the fit of all expression data to count data showed high correlation (F: R2=0.79). 

## Data Availability

All data generated in this study are available within the paper and its additional files. The raw sequence data reported in the paper have been deposited in NCBI as BioProject: PRJNA743032.

## Abbreviations

QTL: Quantitative trait loci
NIL: Near isogenic line
DEG: Differentially expressed gene
CB: California Blackeye
EPG: Electrical penetration graph
CNL: Coiled-coil nucleotide-binding site-leucine rich repeat
ROI: Region of interest
RLK: Receptor-like kinase
