# Supplementary material for: Transcriptome analysis of aphid-resistant and susceptible near isogenic lines reveals candidate resistance genes in cowpea (Vigna unguiculata)
Source: BMC Plant Biol. 2023 Jan 11;23:22. doi: 10.1186/s12870-022-04021-w (PMC9832699; doi:10.1186/s12870-022-04021-w)
Supplement: Supplementary file 1 — Additional file 1: Figure S1. Damage induced by cowpea aphids after 7-days feeding on susceptible cowpea results in chlorosis and necrosis near feeding sites and leaf curling. Photos depict week-old plants infested with 15 adult apterous aphids. Figure S2. Growth of cowpea aphid population on susceptible (CB46) and resistant (CB77) cowpea. Two-week-old plants were infested with 20 adult apterous aphids on a single unifoliate leaf and enclosed in a mesh sleeve bag for 1 week. The aphid population was counted daily. Data from MacWilliams et al. 2022. Figure S3. Gene ontology (GO) analyses indicated numerous processes enriched in resistant and susceptible plants over time (day 6 vs day 1) that may play a role in constitutive resistance. Grey shading indicates processes uniquely expressed within each genotype. Figure S4. Gene ontology (GO) analyses indicated numerous processes are enriched when cowpea aphids feed on susceptible plants, with more processes upregulated than downregulated after correcting for leaf development. Resistant plants show enrichment in only 20% (6/30) of the total pathways enriched in susceptible plants when aphids feed. Figure S5. MDS plots for RNAseq show separation of time points. Overall, each treatment clusters together with the largest separation explained by time. Figure S6. qRT-PCR validation of select genes differentially expressed in RNAseq data relative to susceptible (CB46) uninfested plants. Genes consistently upregulated across time in resistant cowpea (CB77; A:RGA4-600, B:RGA4-700) and genes differentially expressed among treatments (C:CHiB, D:STE11) showed expression trends in concordance with RNAseq data (*) relative to susceptible control tissues at the collection time point. Primers for select genes are listed (E) and the fit of all expression data to count data showed high correlation (F: R2=0.79). [file 12870_2022_4021_MOESM1_ESM.pdf]

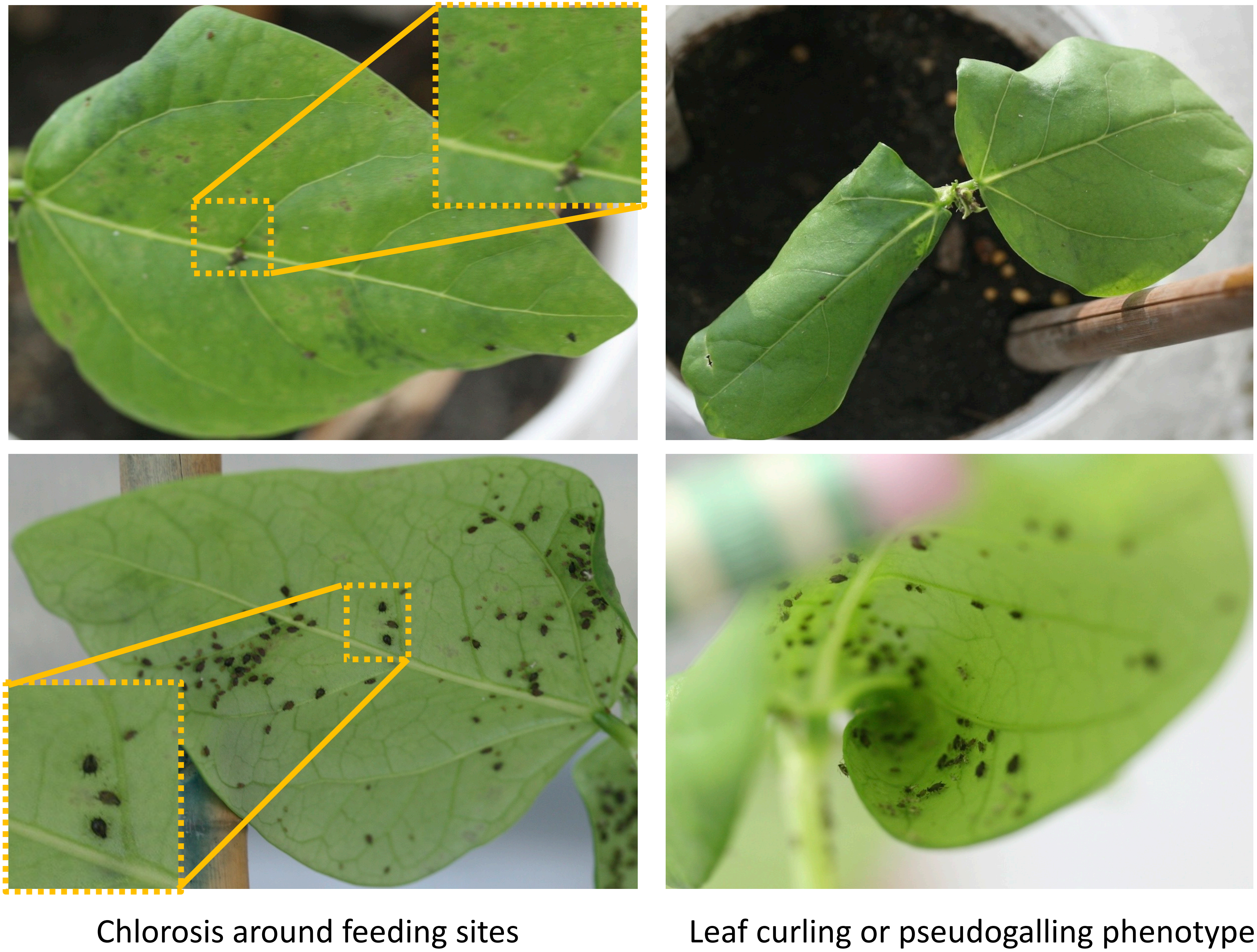

Figure S1 Damage induced by cowpea aphids after 7-days feeding on susceptible cowpea results in chlorosis and necrosis near feeding sites and leaf curling. Photos depict week-old plants infested with 15 adult apterous aphids.

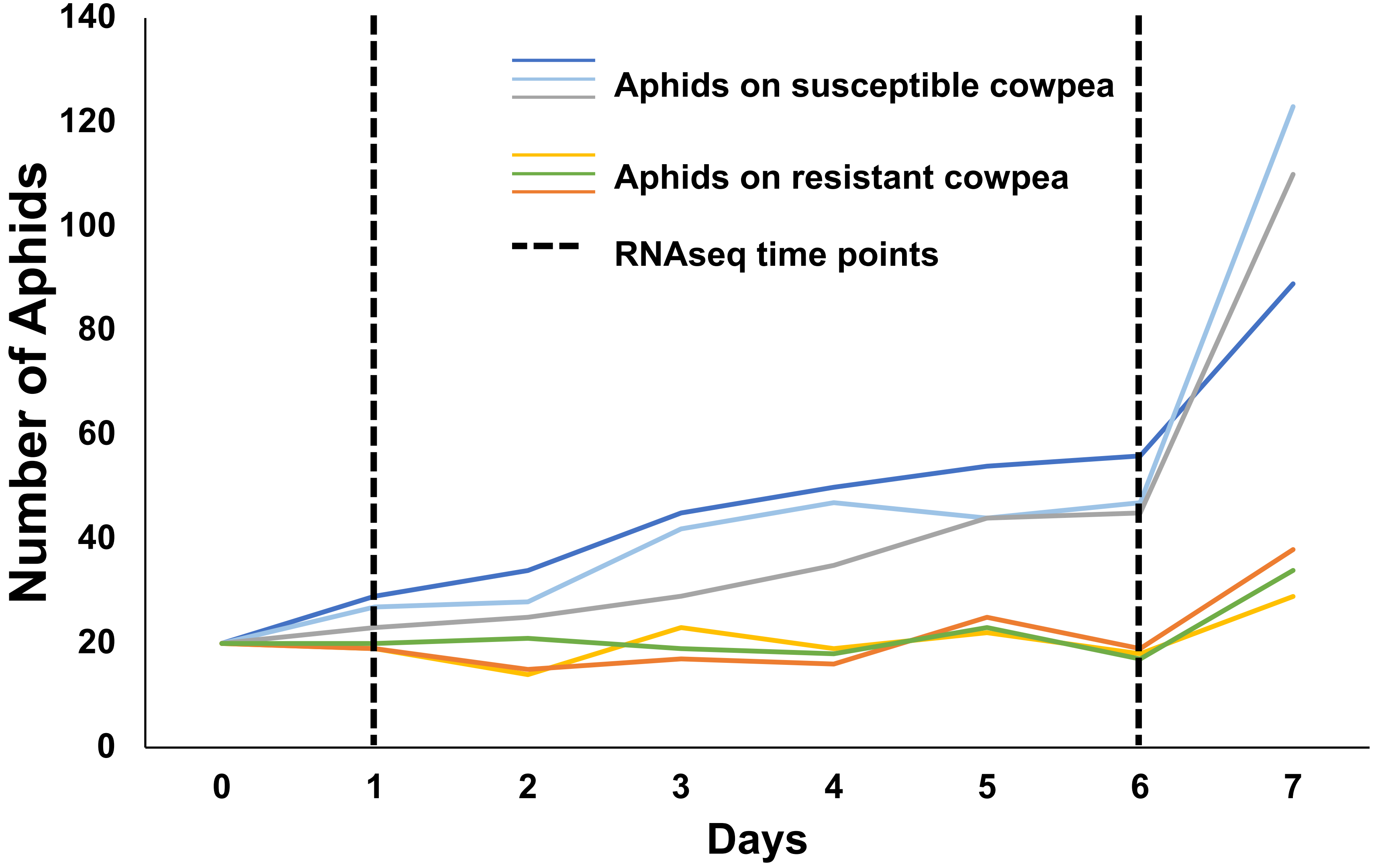

Figure S2. Growth of cowpea aphid population on susceptible (CB46) and resistant (CB77) cowpea. Two-week-old plants were infested with 20 adult apterous aphids on a single unifoliate leaf and enclosed in a mesh sleeve bag for 1 week. The aphid population was counted daily. Data from MacWilliams et al. 2022.

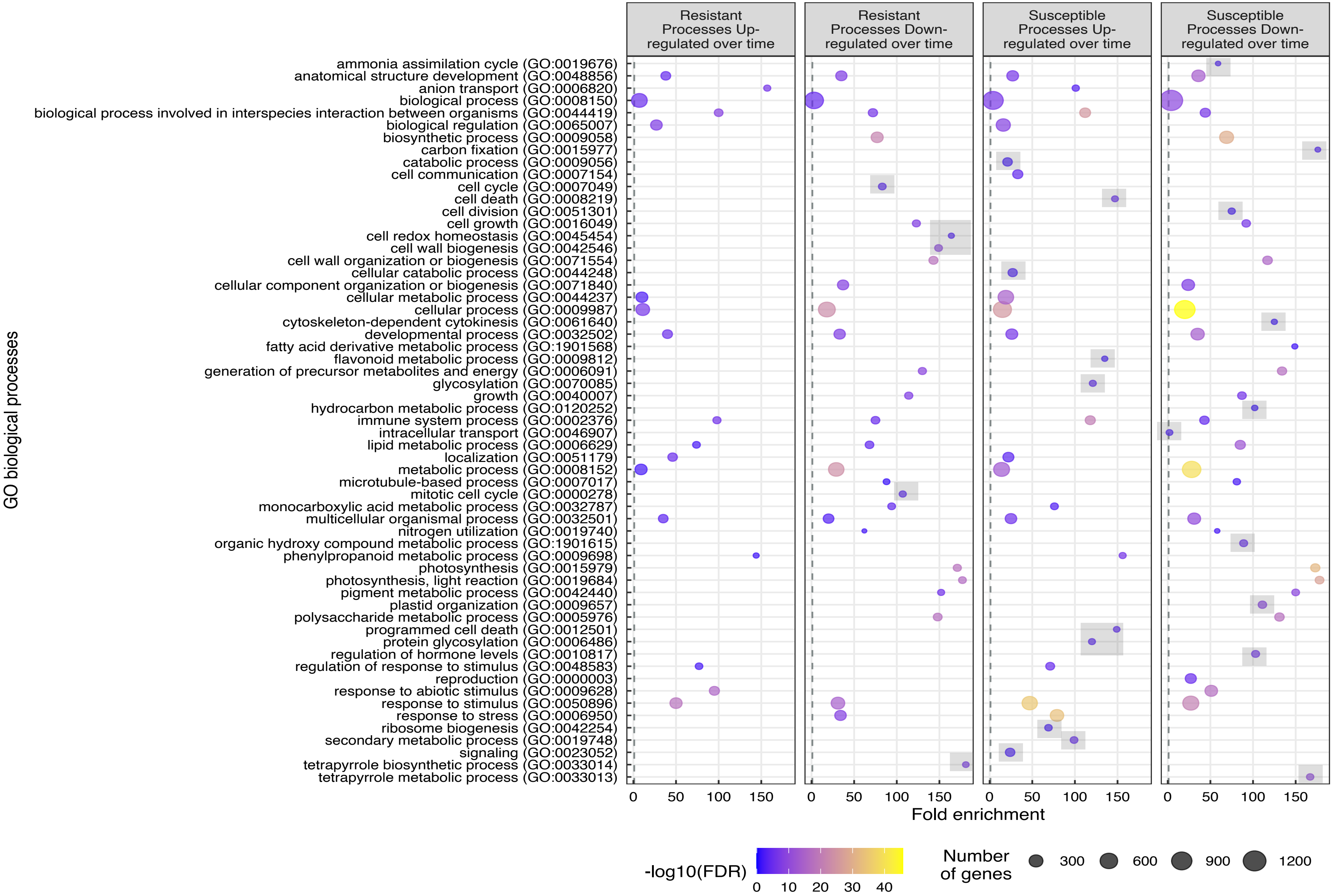

Figure S3. Gene ontology (GO) analyses indicated numerous processes enriched in resistant and susceptible plants over time (day 6 vs day 1) that may play a role in constitutive resistance. Grey shading indicates processes uniquely expressed within each genotype.

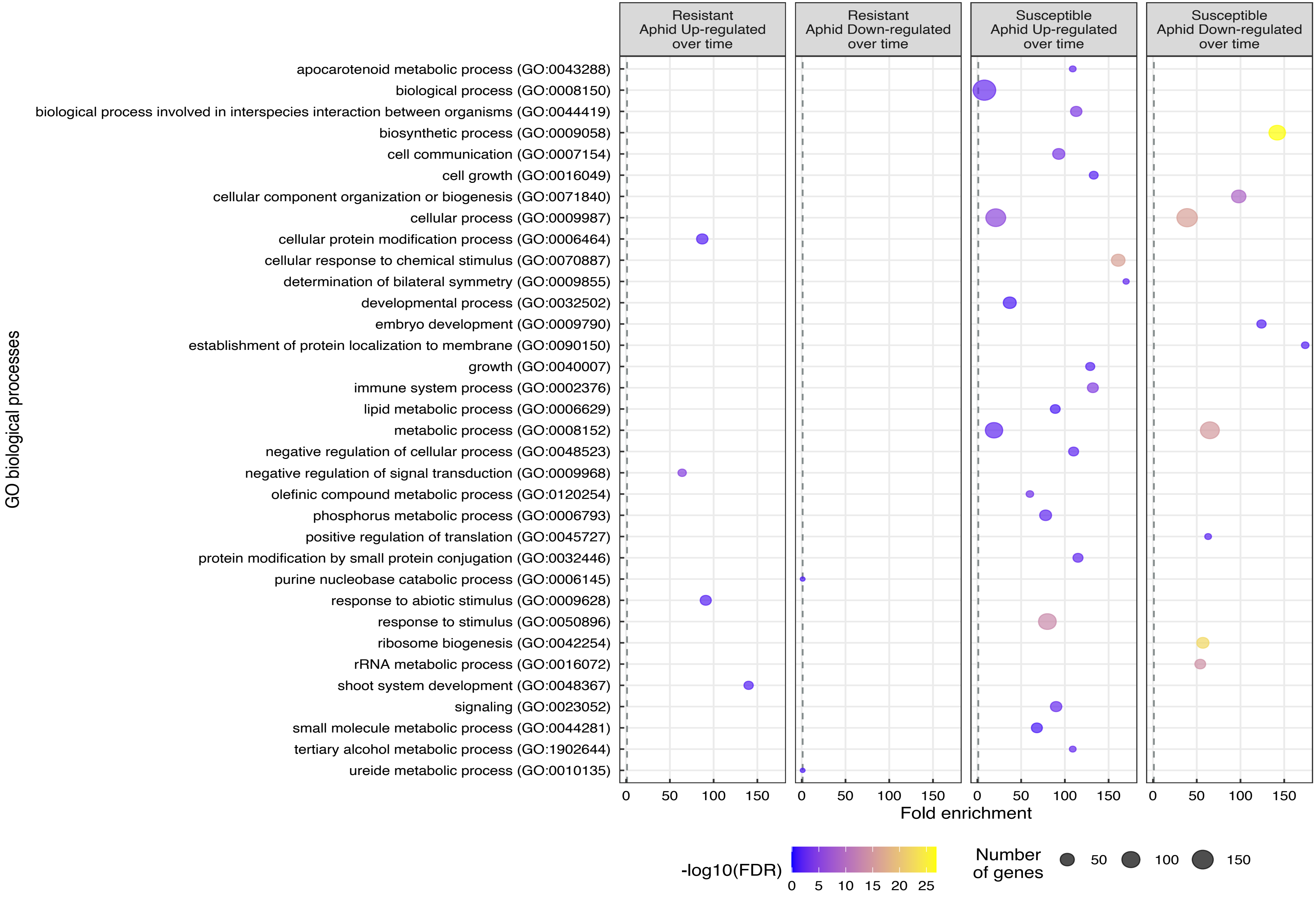

Figure S4. Gene ontology (GO) analyses indicated numerous processes are enriched when cowpea aphids feed on susceptible plants, with more processes upregulated than downregulated after correcting for leaf development. Resistant plants show enrichment in only 20% (6/30) of the total pathways enriched in susceptible plants when aphids feed.

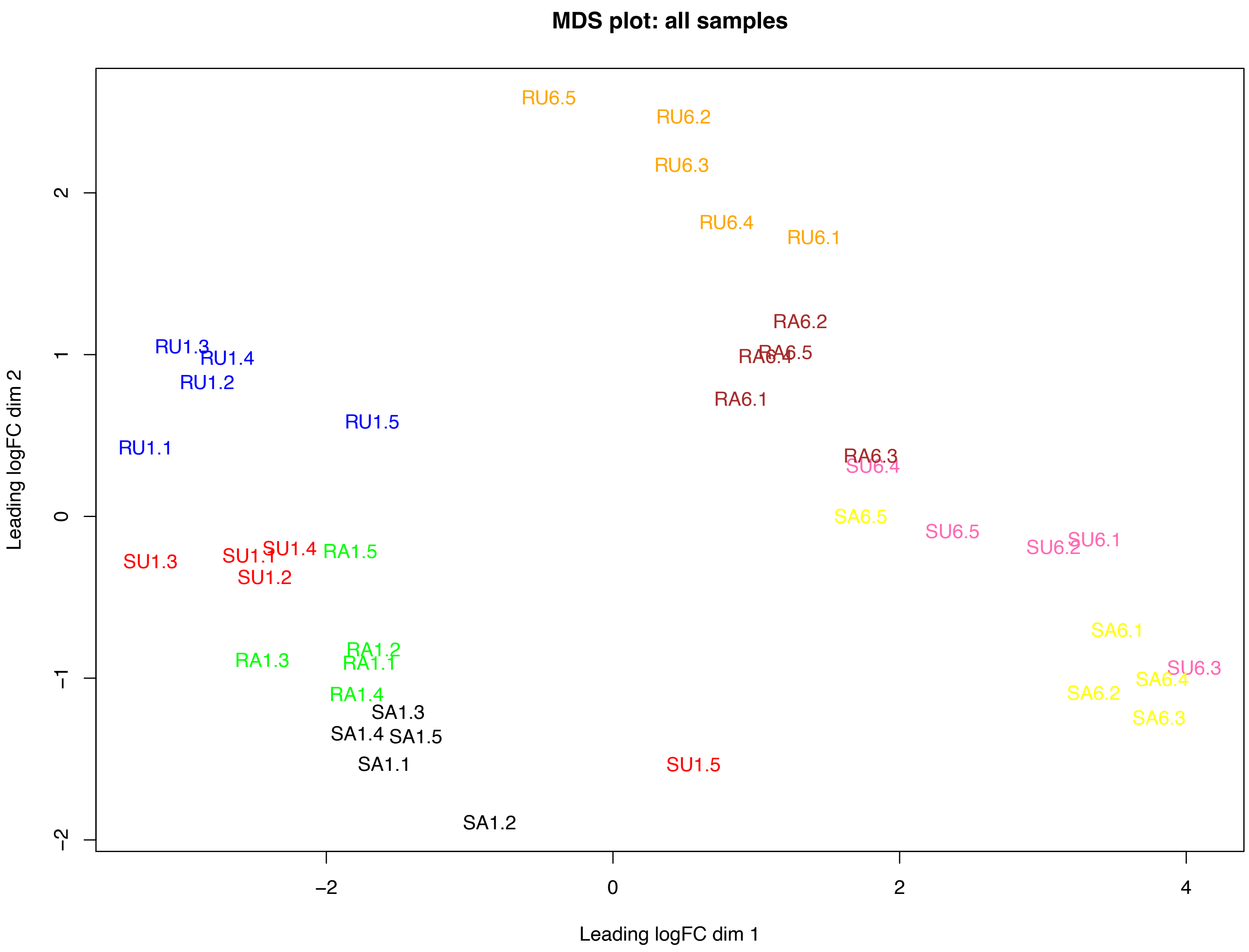

Figure S5. MDS plots for RNAseq show separation of time points. Overall, each treatment clusters together with the largest separation explained by time . Treatments are denoted by Susceptible (S), Resistant (R), Aphid-infested (A), Un-infested (U) and day 1 or 6, i.e., RA1 is Resistant genotype infested by aphids for 1 day.

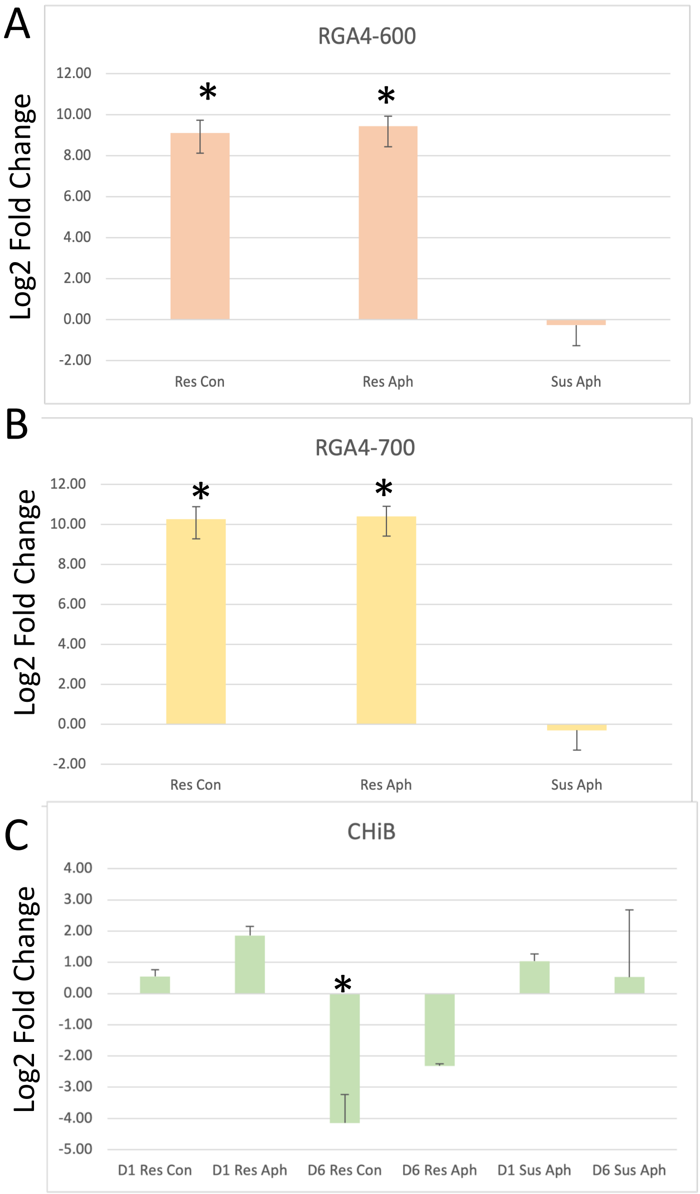

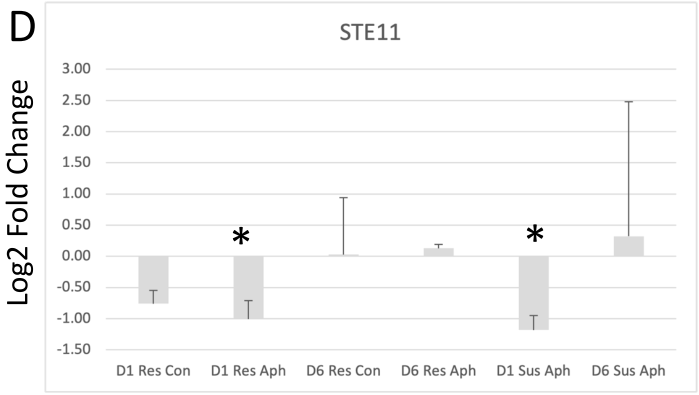

E

Gene	***Vigna unguiculata* 1.2 ID**	**Forward**	**Reverse**
VuRGA4-700	Vigun02g100700.1	CAGCACGTGGCAATCATTTATTGC	GACAAGTTGGTGAGGCGTTGAATGC
VuRGA4-600	Vigun02g100600.1	CAGCACGTCTCAATCAATATTTGC	GACAAGTTGGTGAGGCGTTGAATGC
VuCHiB	Vigun09g278000	CCATCTGGTTCTGGATGACC	CCGTTGATGATGTTCGTCAC
VuSTE11	Vigun03g278000	TCCACTGCGACATAAAGCTG	CCCTGCATTCACTCTTCCTC
VuUbq11	Vigun02g198500.1	GAGCTCAAGGACCTCCAGAA	CTAGAAAAACACCCCAGCA

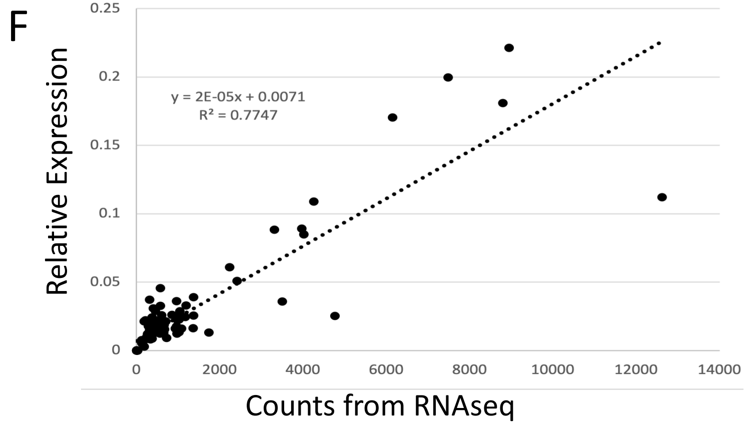

Figure S6. qRT-PCR validation of select genes differentially expressed in RNAseq data relative to susceptible (CB46) uninfested plants. Genes consistently upregulated across time in resistant cowpea (CB77; A:RGA4-600, B:RGA4-700) and genes differentially expressed among treatments (C:CHiB, D:STE11) showed expression trends in concordance with RNAseq data (*) relative to susceptible control tissues at the collection time point. Primers for select genes are listed (E) and the fit of all expression data to count data showed high correlation (F: R^2=0.79).
